# Natural Flavonoids Derived From Fruits Are Potential Agents Against Atherosclerosis

**DOI:** 10.3389/fnut.2022.862277

**Published:** 2022-03-24

**Authors:** Ruo-Lan Li, Ling-Yu Wang, Shuqin Liu, Hu-Xinyue Duan, Qing Zhang, Ting Zhang, Wei Peng, Yongliang Huang, Chunjie Wu

**Affiliations:** ^1^State Key Laboratory of Southwestern Chinese Medicine Resources, School of Pharmacy, Chengdu University of Traditional Chinese Medicine, Chengdu, China; ^2^Hospital of Chengdu University of Traditional Chinese Medicine, Chengdu, China

**Keywords:** natural flavonoids, fruits, atherosclerosis, cardiovascular diseases, potential mechanism

## Abstract

Atherosclerosis, as a chronic inflammatory response, is one of the main causes of cardiovascular diseases. Atherosclerosis is induced by endothelial cell dysfunction, migration and proliferation of smooth muscle cells, accumulation of foam cells and inflammatory response, resulting in plaque accumulation, narrowing and hardening of the artery wall, and ultimately leading to myocardial infarction or sudden death and other serious consequences. Flavonoid is a kind of natural polyphenol compound widely existing in fruits with various structures, mainly including flavonols, flavones, flavanones, flavanols, anthocyanins, isoflavones, and chalcone, etc. Because of its potential health benefits, it is now used in supplements, cosmetics and medicines, and researchers are increasingly paying attention to its role in atherosclerosis. In this paper, we will focus on several important nodes in the development of atherosclerotic disease, including endothelial cell dysfunction, smooth muscle cell migration and proliferation, foam cell accumulation and inflammatory response. At the same time, through the classification of flavonoids from fruits, the role and potential mechanism of flavonoids in atherosclerosis were reviewed, providing a certain direction for the development of fruit flavonoids in the treatment of atherosclerosis drugs.

## Introduction

Cardiovascular disease (CVD) is a kind of disease with extremely high morbidity and mortality. According to relevant investigations, CVD deaths accounted for about 31% of global deaths in 2016, among which atherosclerosis is the main cause of CVD (1). Atherosclerosis is a chronic inflammatory disease, mostly affecting adults and the elderly. It is characterized by plaque accumulation, narrowing and hardening of coronary artery walls, which will directly affect the completion of blood oxygen supply to various organs in the body, resulting in serious consequences such as myocardial infarction, angina pectoris and sudden death (2, 3). The pathogenesis of atherosclerosis is diverse. In current studies, the factors that affect atherosclerosis are mainly hyperlipidemia, diabetes, smoking, high blood pressure, genetic, and other cardiovascular risk factors. These factors can induce dysfunction of endothelial cells through mediating oxidative stress, and then leads to the beginning of the atherosclerotic disease process (4).

At present, pharmacologic treatment with medications, stent-based therapy or coronary artery bypass surgery are commonly used in clinical treatment of atherosclerosis to relieve symptoms, but three methods have certain limitations (5, 6). For example, statin is a widely used drug in clinical practice, which can inhibit the occurrence and development of atherosclerosis by inhibiting cholesterol synthesis. However, due to its poor targeting, oral or intravenous administration can also attack normal tissues and cells, resulting in strong side effects. In addition, when the disease develops to an advanced stage, drug treatment is less effective (7). Although coronary artery bypass surgery can significantly reduce the mortality of patients with atherosclerosis, its prognosis is poor and it is easy to cause various complications (6). Stent-based therapy can also help relieve patients’ related symptoms and have a low incidence of disease complications in the advanced stage of the disease when drugs fail to play a role. But it’s a pity that problems such as artery stenosis, inflammation and thrombosis in patients with stent treatment have not been solved, so the treatment can only relieve their symptoms but not solve their causes (5). Therefore, it is urgent to find new compounds for the treatment of atherosclerosis.

Flavonoids are a kind of natural organic compounds widely present in fruits, which are composed of two aromatic rings and have typical C6-C3-C6 skeleton (8). Previous studies have found that a diet rich in flavonoids can significantly reduce CVD mortality, which is directly related to atherosclerosis. At the same time, the effect is related to the source, dose and bioavailability of flavonoids (9). The current pharmacological studies have showed that a variety of flavonoids from fruits could not only reduce cholesterol transport, but also enhanced the immune function by regulating the level of intracellular inflammatory factors (10). In addition, hydroxyl radicals, which are widely present in flavonoids, also play a role in protecting blood vessels by mediating antioxidant effects (11). Epidemiological studies linking flavonoid intake to a reduced risk of death from CVD have generated considerable interest in this preventive mechanism (12). As fruit is the most important component in the source of flavonoids, the treatment of atherosclerosis by flavonoids derived from fruit will be reviewed in this paper.

## Atherosclerosis

Due to the different components, the arteries can be classified as elastic arteries, muscular arteries, and transitional regions between the two kinds of arteries. The artery wall has three layers of tissue structure, of which the most inward layer is composed of endothelial cells, known as the intima. The outermost layer is composed of connective tissues, collagen, and elastic fibers, while the medial membrane is composed of vascular smooth muscle cells (VSMCs) (13). As a chronic inflammatory response, atherosclerosis is at increased risk for environmental and genetic factors. In the early stage of atherosclerotic disease, hypercholesterolemia induces the entry of low-density lipoprotein (LDL) into the subcutaneous space of intima and promotes the oxidation of LDL under enzymatic or non-enzymatic modification, thereby activating endothelial cells and causing endothelial dysfunction (14). In particular, activated endothelial cells attract monocytes and other white blood cells by upregulation of adhesion molecules and secretion of chemokines, which ultimately lead to chronic inflammatory responses (15, 16). During the development of atherosclerosis, monocytes differentiate into macrophages and phagocytose oxidized low-density lipoprotein (ox-LDL) to form foam cells. Subsequently, foam cells can attract VSMCs to migrate to the subcutaneous space and proliferate, resulting in the formation of new intima in the arterial lumen and leading to arterial narrowing (17). As the inflammatory response within the arterial vasculature continues to occur and the lumen becomes progressively narrower, the arterial vasculature is highly susceptible to rupture and subsequent thrombosis, which can lead to more serious clinical complications. Therefore, in this section, we will focus on an overview of several important points in the development of atherosclerotic disease, namely, endothelial dysfunction, foam cell formation, migration, and proliferation of VSMCs and inflammatory response.

### Endothelial Dysfunction

Vascular endothelial cells are epithelial cells arranged in a single layer on the inner side of blood vessels with a large surface area and at a critical location where blood circulation and tissue intersection (18). They have multiple physiological functions. Functioning endothelial cells can effectively regulate vascular permeability and vascular tension, and also be used as active signal transducers for circulating influences that modify the vessel wall phenotype (19). However, when endothelial cells encounter shear stress, dyslipidemia, hyperglycemia, aging and other factors, endothelial cell dysfunction and vascular homeostasis disorders, which then lead to a series of consequences such as vasoconstriction, leukocyte adherence, platelet activation, and promotion of oxidation, and ultimately lead to atherosclerosis (20). Continuous DNA replication, oxidative stress, and mitochondrial dysfunction may exert pressure on cells to permanently inhibit proliferation and lead to cell senescence (21). In response to this stress, cells secrete a variety of proteins named senescence-associated secretory phenotype (SASP), including pro-inflammatory cytokines (interleukin-6, interleukin-8, macrophage inflammatory proteins, etc.), chemokines, growth factors, matrix metalloproteinases, and other signaling molecules. There is no doubt that the transient expression of these proteins will repair the damaged tissue, but when the body is exposed to this environment for a long time, it will accelerate endothelial dysfunction (22–24). In addition to aging, dyslipidemia is another important cause of endothelial cell dysfunction. When the level of serum high-density lipoprotein (HDL) decreases and the level of total cholesterol (TC), triglyceride (TG), and low-density lipoprotein cholesterol (LDL-C) increases, LDLs will accumulate in the subcutaneous space of the artery wall and oxidize to form oxLDL under enzymatic or non-enzymatic modification (25). It further promoted the expression of monocyte chemotactic protein 1 (MCP-1), vascular cell adhesion molecule-1 (VCAM-1), endothelial leukocyte adhesion molecule (E-selectin), and finally induced inflammatory response (26, 27).

**FIGURE 1:**
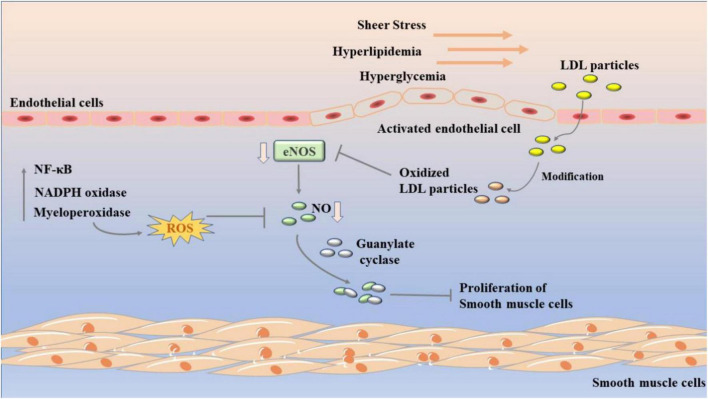
Development of endothelial dysfunction in atherosclerosis. Sheer stress, hyperlipidemia, and hyperglycemia leads to endothelial dysfunction. LDLs will accumulate in the subcutaneous space of the artery wall and oxidize to form oxLDL. It further decreases the activity of eNOS, which in turn reduce the content of NO. The nuclear transcription factor kappa B (NF-κB), NADPH oxidase (NOX), and myeloperoxidase (MPO) are related to its process.

Endothelial dysfunction is characterized by endothelium-dependent vasodilation injury and endothelial activation marked by proinflammatory, proliferative, and procoagulant states, in which disruption of nitric oxide (NO) bioavailability is central (28). NO is a major vasodilator. Due to its small molecular weight, NO can diffuse to VSMC to activate guanylate cyclase, leading to cGMP-mediated vasodilation. At the same time, it can also spread to vascular lumen to inhibit platelet aggregation and adhesion, thus achieving anti-thrombotic effect (29, 30). In addition, as shown in Figure 1, NO can inhibit vascular smooth muscle proliferation by inhibiting platelet and leukocyte activation. However, when endothelial cells are activated, the production of endothelial NO synthase (eNOS) from L-arginine is reduced and tetrahydrobiopterin is absent, leading to NOS uncoupling and the generation of reactive oxygen species (ROS) such as superoxide and hydrogen peroxide (31, 32). Thus, endothelial cells switch from NO signal to ROS-mediated oxidative stress signal, activating the nuclear transcription factor kappaB (NF-κB) and other signaling pathways (33). In atherosclerosis, ROS production is associated with NADPH oxidase (NOX), myeloperoxidase (MPO), eNOS, and lipoxygenase. Of course, in addition to NO, prostacyclin prostacyclin (PGI2) and endothelium-derived hyperpolarizing factor (EDHF) and other vasodilators maintain vascular motility together with endothelin-1 (ET-1) and angiotensin II (AngII) (34–36).

### Formation of Foam Cells

Foam cells are a group of cells with multiple lipid inclusions in the cytoplasm. Most of them exist in the lipid rich endothelial space beneath the arteries. The appearance of foam cells is often regarded as one of the early manifestations of atherosclerosis (37). In the current study, it is generally believed that foam cells are mainly derived from macrophages, endothelial cells and VSMCs, and are mostly combined with modified LDL and cholesteryl ester (CE) after macrophages pass through the endothelial barrier (38). According to relevant data, 90% of macrophages in the artery are located in the adventitial layer, only 10% are located in the intima. Besides this, macrophages in the intima are almost formed only after birth (39, 40). Hypercholesterolemia is often accompanied by persistent inflammation, endothelial cell activation and secretion of chemokines such as CCL2/MCP-1, CX3CL1, and CCL5. This phenomenon will cause a large number of monocytes recruit to the area of LDL modification and promote the differentiation of monocytes into macrophages, which can quickly recognize and absorb modified LDL into foam cells (41–43). Foam cell formation is a complex process which is affected by many factors. Although the accumulation of lipid in macrophages is mainly derived from modified LDL, unmodified LDL in blood does not induce foam cell formation under normal physiological conditions. During the development of atherosclerosis, LDL will undergo a variety of modifications such as oxidation, carbamylation, and glycosylation to change its characteristics, so that it can be recognized and absorbed by macrophages (44).

**FIGURE 2:**
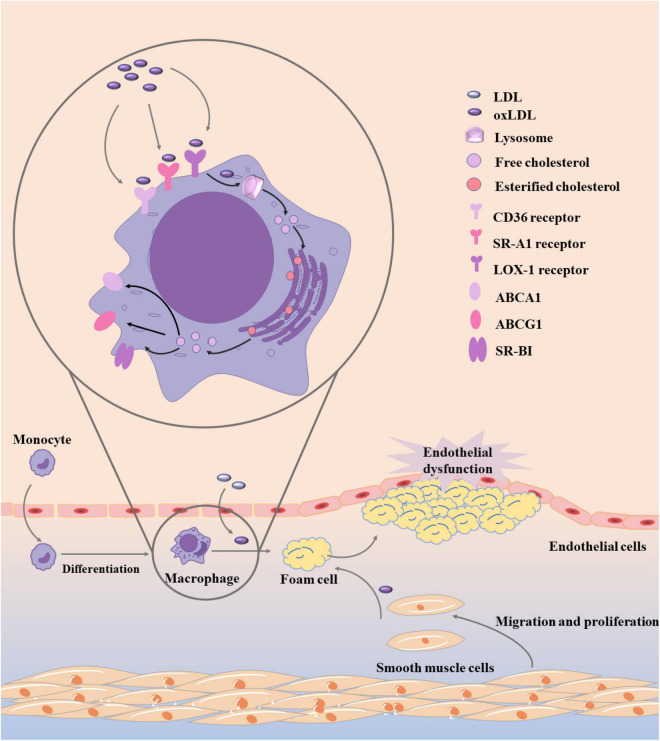
Development of foam cells in atherosclerosis. Monocytes recruit to the area of LDL modification and differentiate into macrophages, which can quickly recognize and absorb modified LDL into foam cells. Besides the modified LDL, the disorder of lipid metabolism in macrophages is another important factor of foam cell production, and the homeostasis of lipid metabolism in macrophages is mainly coordinated by three main processes, including cholesterol uptake, cholesterol esterification, and cholesterol efflux.

Besides the modified LDL, the disorder of lipid metabolism in macrophages is another important factor of foam cell production (45). As shown in Figure 2, the homeostasis of lipid metabolism in macrophages is mainly coordinated by three main processes, including cholesterol uptake, cholesterol esterification, and cholesterol efflux (45). The imbalance of any of the three processes may lead to the increase of foam cells. Cholesterol uptake in macrophages mainly recognizes and absorbs modified LDL through a variety of scavenging receptors. CD36 is a glycoprotein that can promote cholesterol uptake. Its high expression often follows the emergence of ox-LDL (46, 47). Therefore, CD36 is often used as a biomarker of atherosclerosis in modern diagnosis and treatment. In macrophages, the expression of CD36 is often activated by peroxisome proliferator-activated receptors-γ (PPAR γ), nuclear erythroid-related factor 2 (Nrf2), signal transducer and activator of transcription (STAT) 1, and activator protein-1 (AP-1) are regulated (48, 49). When the expression of CD36 was inhibited, cholesterol uptake was significantly reduced and the symptoms of atherosclerosis were alleviated. In addition to CD36, scavenging receptor A1 (SR-A1) and lectin like ox-LDL receptor-1 (LOX-1) can also promote the recognition and absorption of modified LDL, while LOX-1 is the main receptor for endothelial cells to bind ox-LDL (50). What’s more, in macrophages, the expression of SR-A1 is regulated by NF-κB, AP-1, and PPAR γ, while the expression of LOX-1 is mainly regulated by NF- κB, AP-1, and POU-domain transcription factors (45, 51).

After the modified LDL was recognized and absorbed by macrophages, it was first transformed into free cholesterol by lysosomal acid lipase (LAL) in lysosome. When cholesterol accumulates excessively, cholesterol acyltransferases-1 (ACAT1) and –2 (ACAT2) in the endoplasmic reticulum will esterificate free cholesterol again. Subsequently, cholesteryl ester hydrolases (CEH) such as hormone sensitive lipase (HSL), carboxyl ester lipase (CEL), and neutral cholesterol ester hydrolase 1 (NCEH1) can hydrolyze esterified cholesterol again (52–54). During this process, the esterification and hydrolysis of cholesterol should be balanced. If the balance is broken, the generation rate of foam cells will be accelerated. The re-esterification of cholesterol in macrophages can prevent the accumulation of free cholesterol from damaging cells, but this process has a certain limit. When the cholesterol exceeds a certain range after re-esterification, a large number of lipid droplets will be generated in cells (55).

Of course, the content of free cholesterol in macrophages should not exceed the limit. In addition to the re-esterification mentioned above, free cholesterol can also maintain intracellular metabolic balance through cholesterol efflux process. ATP-binding cassette transporter A1 (ABCA1), ATP-binding cassette transporter G1 (ABCG1), and scavenger receptor class B type 1 (SR-BI) are mainly involved in the process of cholesterol efflux, which can bind to free cholesterol and transport out of cells (56). Subsequently, ABCA1 carrying cholesterol preferentially bind to apolipoprotein A1 (apoA1) to produce HDL particles, ABCG1 preferentially interacts with mature HDL particles, and SR-BI interacts with a variety of lipoproteins (45, 57). PPAR γ, liver X receptor (LXR), retinoid X receptor, and some miRNAs can regulate the expression of ABCA1, ABCG1, and SR-BI (58, 59).

### Migration and Proliferation of Vascular Smooth Muscle

Vascular smooth muscle cells are the most abundant cell type in the arterial wall, and have phenotypic plasticity. They can show different phenotypes in different arteries or different diseases (60). In healthy blood vessels, VSMCs can maintain homeostasis by adjusting their phenotypes to adapt to changes in blood flow when hemodynamics changes. Conversely, when arteries become diseased, this ability was reduced and homeostasis was broken, which could exacerbate the disease (61). Mature VSMCs, for example, have a low proliferation rate and can respond to changes of NO and ET-1 from endothelium and regulate blood flow by regulating blood vessel diameter through contraction (62). Unfortunately, because VSMCs are not in a final differentiation state, when atherosclerosis occurs, the expression of specific markers of mature VSMCs under biochemical and biomechanical stimulation is inhibited, a large number of VSMCs differentiate into synthetic phenotypes and migrate to the intima of arterial wall under the guidance of platelet-derived growth factor B (PDGF-B) (63). On the one hand, activated VSMCs proliferate in the intima and narrow the arterial lumen, which are regarded as the main features of atherosclerosis. On the other hand, VSMCs produce collagen fibers and elastic fibers under the stimulation of transformational growth factor-β (TGF-β), change the composition of extracellular matrix and envelops lipids by fiber caps to form typical atherosclerotic plaques (64). In addition, the latest research also shows that fibro-myocytes differentiated by VSMC can stabilize the plaque, and when they differentiate into cartilage, osteoblasts or inflammatory cells, they can aggravate the development of atherosclerosis (65, 66). Thus, the phenotypic transformation of VSMCs is crucial in atherosclerosis.

**FIGURE 3:**
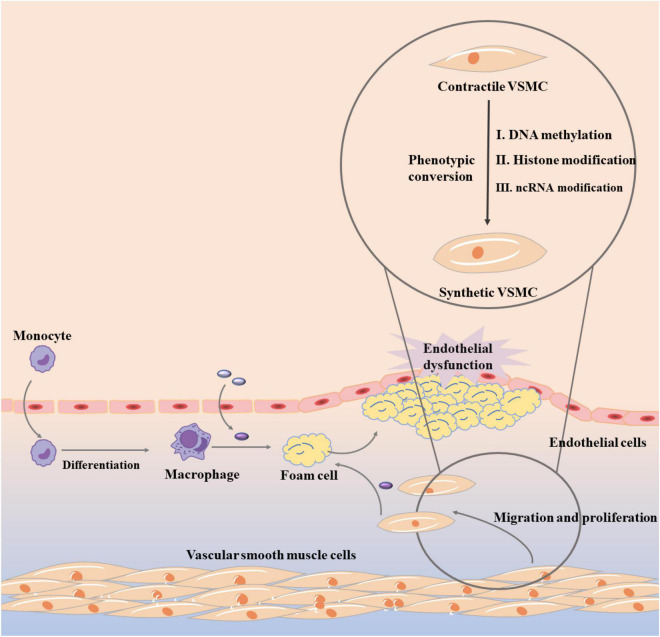
Vascular smooth muscle cell phenotypic conversion in atherosclerosis.

In the past few decades, increasing experiments have focused on the process of controlling VSMC phenotypic conversion, but the key molecular mechanism has not been clearly clarified (67). Subsequently, there are growing evidences that epigenetic mechanisms provide transcriptional control that can directly cause phenotypic switch in VSMC, which is shown in Figure 3 (68). Theoretically, epigenetic mechanism is to change gene expression through three main epigenetic modifications, DNA methylation, histone modification, and non-coding RNA (ncRNA) modification without changing the genome (69, 70). In atherosclerosis, DNA methylation can regulate a variety of genes that define VSMC phenotypic transformation, such as serum response factor (SRF), PDGF-B, and TAGLN (64). DNA methyltransferase 1 (DMNT1) and Ten-eleven translocated methylcytosine deoxygenase 2 (TET2), two major enzymes that control DNA methylation, also play an important role (71). It was found that knockdown of TET2 suppressed the expression of key VSMC genes such as MYOCD and SRF, while transcriptional upregulation of KLF4 initiated VSMC phenotypic transition (72). When TET2 was overexpressed, VSMC phenotype conversion was inhibited and intimal hyperplasia was significantly improved. In contrast, when DNMT1 was repressed, MYOCD expression was increased and VSMC phenotypic conversion was inhibited (73). Histone modification mainly includes methylation, acetylation, and ubiquitination. The role of histone methylation and acetylation in atherosclerosis and VSMC phenotype transformation cannot be ignored, and most of them appear in a combination form (74). For example, a significant decrease in H3K9 and H3K27 methylation and a significant increase in H3K9 and H3K27 acetylation levels were observed in atherosclerotic plaques (75, 76). Besides, VSMC phenotypic transition can be regulated by microRNAs (miRNAs) and long-stranded non-coding RNA (lncRNAs) (77).

### Inflammation

As we all know, atherosclerosis is a chronic inflammatory disease. Inflammation is accompanied by the initiation and development of the whole disease. After decades of extensive research, we have preliminarily elucidated that the related inflammatory response in atherosclerosis is mediated by proinflammatory cytokines, adhesion molecules, inflammatory signaling pathways and bioactive lipids (78). In general, healthy endothelial cells are able to effectively resist leukocyte adhesion, and acute inflammation can restore normal tissue structure through leukocyte infiltration and subsequent clearance mechanism (79). However, when related events such as hypertension and hyperglycemia occur, endothelial cells activate and consequently express monocyte chemoattractant protein-1, interleukin (IL)-8, intercellular adhesion molecule-1 (ICAM-1), vascular adhesion molecule-1 (VCAM-1), E-selectin, P-selectin, and other inflammatory factors, resulting in monocyte retention and triggering chronic inflammatory injury (80). This view was further proved *in vitro* experiments. Pro-inflammatory monocytes with high expression of Ly6C preferentially adhere to cytokine-stimulated endothelial cells, and dendritic cells, T cells and neutrophils are also involved in this inflammatory response (81). With the development of atherosclerosis, macrophages, VSMC, and endothelial cells in arteries can secrete a variety of matrix metalloproteinases (MMPs). MMP-9 can increase macrophage infiltration and collagen deposition, while MMP-2 can promote extracellular matrix degradation and VSMC migration (82, 83). Both of them work together to form an arterial pro-inflammatory environment and aggravate the inflammatory reaction in atherosclerosis.

**FIGURE 4:**
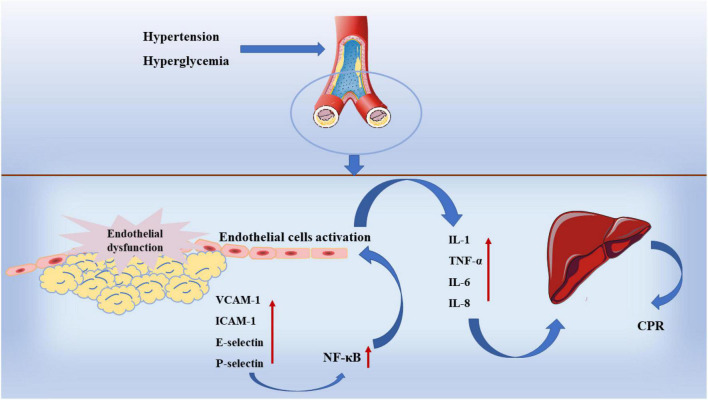
Inflammation in atherosclerosis.

In fact, although inflammatory response is involved in all processes of atherosclerosis, there is no practical evidence to support the inflammatory hypothesis in early studies until the discovery of inflammatory markers (84). In addition to MMPs, IL-6, C-Reactive protein (CRP), and adhesion molecules are inflammatory markers. When the inflammatory response is turned on in arteries, macrophages and adipocytes release large amounts of IL-6 and TNF- α,inducing a downstream inflammatory cascade to occur (85). In another experiment, it was also found that the risk of coronary heart disease increased with the upregulating of IL-6 level in plasma and was positively correlated with the severity of the disease (86). At the same time, the release of IL-6, IL-1β, and TNF-α stimulated the synthesis of CRP in the liver and adipose tissue, prevents the proliferation and repair of vascular endothelial cells (87). Selectin family, immunoglobulin superfamily (IgSF) and integrin family of adhesion molecules are involved in the development of atherosclerosis (88). Among them, vascular cell adhesion molecule-1 (VCAM-1) and ICAM-1 play an important role. As shown in Figure 4, VCAM-1 can activate endothelial cells by upregulating the transcription factor nuclear factor-κB (NF-κB), which causes endothelial cells to release various pro-inflammatory cytokines such as IL-1, TNF-a, IL-6, and IL-8 (89). The high expression of VCAM-1 and ICAM-1 can promote the proliferation of macrophages, lead to the excessive accumulation of macrophages in the plaque and reduce the stability of the plaque (90). At the same time, VCAM-1 and ICAM-1 can also promote the formation of high permeability and fragile neovascularization (91).

As researchers have progressively studied atherosclerosis, the inflammatory signaling pathways associated with the disease continue to attract more attention. From the known studies, toll like receptor 4 (TLR4), NF- κB, Janus kinase (JAK) signal transducers and activators of transcription (STAT) have been identified as major signaling pathways. ABCG1, a key gene linking lipid accumulation and inflammation, can be regulated by TLR4 in the organism (92). After the onset of atherosclerotic process, TLR4 activates the peroxisome proliferator-activated receptor γ (PPAR-γ)/liver X receptor α (LXRα) signaling pathway, which in turn downregulates ABCG1 expression (93). Meanwhile, TLR4 can also promote the release of MCP-1, IL-1α, and IL-6 by activating NF-κB, which induces lipid accumulation in the arterial vasculature and the development of inflammation (94). In atherosclerotic, JAK/STAT is mainly activated by cytokines of JAK kinases (JAK1, JAK2, JAK3) and tyrosine kinase (Tyk)2. In experiments, it was found that activation of p-STAT3 was often accompanied by elevated levels of IL-6 and TNF-α, while activation of STAT4 similarly caused secretion of IFN-γ and TNF-α, which activated macrophages and made arterial plaques larger (95, 96).

## Flavonoids Derived From Fruits Are Used to Treat Atherosclerosis

### Flavones

Flavones are a kind of compounds existing in nature and fruits, which play an important role in fruit growth, development, and antibacterial activities. In modern pharmacological research, it is found that the flavones are inseparable from the anti-atherosclerotic effect of fruits (97). Apigenin is a kind of typical flavones named 4′,5,7,-trihydroxyflavone. It is widely found in oranges, grapefruit, and other fruits. Apigenin has high biological activity, can play neuroprotection, antioxidant, anti-tumor, and other effects (98). In addition, it has been found in recent studies that apigenin could participate in all stages of atherosclerosis through a variety of mechanisms, so as to play an anti-atherosclerotic role. As mentioned earlier, hyperlipidemia caused by high-fat diet may be an important factor in inducing atherosclerosis. In SD rats fed with high-fat diet, 8.0 g/kg apigenin was given by gavage for 2 weeks. The results showed that after apigenin treatment, the thickening of aortic intima was alleviated, the contents of TC, TG, and LDL-C decreased, and the content of HDL-C increased, indicating that apigenin could reduce the possibility of atherosclerosis by improving hyperlipidemia (99). However, when hyperlipidemia occurred *in vivo*, the expression of LOX-1 in endothelial cells increased, which promoted the binding of endothelial cells to oxLDL, resulting in endothelial dysfunction. Surprisingly, in HUVECs activated by oxLDL, apigenin could alleviate endothelial cell dysfunction by reducing the expression of LOX-1, VCAM-1, and E-selectin (100). Subsequently, glucose-induced HUVECs and HAECs and trimethylamine-*N*-oxide-induced has cells were used to study the underlying mechanism (101–103). The results showed that apigenin could protect endothelial cells through a variety of signal pathways. For example, apigenin could inhibit endothelial cell apoptosis by decreasing the expression of PKCβII and phosphorylation of NF-κB through ROS/caspase-3 and NO signaling pathway (101). Furthermore, apigenin could also improve the uncontrolled vasodilation and enhance the antioxidant activity of endothelial cells by up-regulating the activity of eNOS and the content of NO and SOD (102, 103). Apigenin also plays an important role in the formation of foam cells. For example, *in vitro* experiments, apigenin could enhance the expression of ABCA1 by inhibiting miR-33, promote the cholesterol efflux in macrophages, and effectively reduce the content of TC, FC and CE in foam cells (104). *In vivo*, apigenin was used to treat LPS-induced ApoE^–/–^ mice, and the same results were obtained. That is, apigenin affected the expression of miR-33, ABCA1, NF-κB p65, and TLR-4, promoting cholesterol efflux and reducing the number of macrophages and smooth muscle cells in atherogenesis, which leads to the decrease in foam cells as well (104). Of course, in addition to affecting lipid metabolism, apigenin could also down-regulate the expression of PAI-2 by inhibiting the phosphorylation of Akt at ser473 site, increase the expression of Bax and cleaved caspase-3 in oxLDL-induced macrophages, and promote macrophage apoptosis (105). In further studies, it was also found that after apigenin treatment, autophagy mediated by ATG5/Atg7 was enhanced in oxLDL-induced macrophages. The role of apoptosis and autophagy accelerates the attenuation of macrophages and relieves the formation of foam cells (106). Simultaneously, apigenin not only inhibited the activation of Caspase-1 by destroying NLRP3 inflammasome assembly, but also reduced mRNA stability by inhibiting ERK1/2 activation in response to inflammation throughout the development of atherosclerosis. The combination of two effects inhibited the secretion of IL-6, IL-1β, and TNF-α, thereby inhibiting the activation of NF-κB in LPS-induced macrophages (107). In addition to apigenin, a variety of flavones derived from fruits in Supplementary Table 1, such as luteolin, tangeretin and chrysoeriol, can inhibit the development of atherosclerosis.

### Flavonols

Flavonols refer to a class of compounds containing 2-phenyl-3-hydroxy (or oxygen-substituted) benzo-γ-pyrone (2-phenyl-3-hydroxy-chromone). They are the most abundant flavonoids, and there are more than 1,700 kinds of flavonols have been found. Quercetin, one of the most abundant flavonols in fruits, has been widely shown to be useful in the prevention and treatment of atherosclerosis. First, quercetin was used to treat Caco-2 cells and human embryonic kidney 293T cells who expressing NPC1L1, and it was found that quercetin inhibited cellular cholesterol uptake by reducing NPC1L1 mRNA levels (132). Subsequently, quercetin was administered to ApoE^–/–^ mice induced by high-fat diet. The results showed that quercetin regulated lipid metabolism by up-regulating the expressions of PPARγ, LXR-α, ABCA1, and down-regulating the expressions of PCSK9 and CD36, reducing the content of TC, LDL-C, oxLDL, and lipid droplets in the cytoplasm, and alleviated the symptoms of atherosclerosis (133). At the same time, quercetin could also reduce the content of TNF-α and IL-6 in the serum of mice, increase the content of IL-10 (133). In another oxLDL-induced RAW264.7 macrophage, quercetin promoted the expression of LC3-II/I and Beclin 1 by reducing the expression of MST1. Simultaneously, quercetin also inhibited the expression of Bcl-2, P21, and P16, which ultimately triggered autophagy in macrophages and reduced foam cell formation (134). Without doubt, quercetin also has excellent efficacy in inhibiting inflammation. In high-glucose-induced human THP-1 monocytic cells, quercetin inhibited the expression of pro-inflammatory genes and related proteins, including TNF-α, IL-1β, COX-2, etc., through the MAPK signaling pathway (115). Correspondingly, in ApoE^–/–^ mice fed on a high-fat diet, quercetin reduced inflammatory by up-regulating Sirt1 and down-regulating Slcam-1 and VCAM-1 expression. All the above results suggest that quercetin is a potential natural compound for the treatment of atherosclerosis (135).

Kaempferol is another flavonol with broad bioactivity that has been shown to reduce the risk of atherosclerosis. Kaempferol was initially confirmed to play a synergistic role with urate in plasma to jointly exert antioxidant effect and reduce oxidative modification of LDL, which preliminarily suggested that kaempferol may have an anti-atherosclerosis effect (136). Subsequently, kaempferol was applied to rabbits fed with a high cholesterol diet. The results showed that after kaempferol treatment, the levels of TNF-α, IL-1β, and MDA in aorta decreased significantly, and the activity of SOD in serum increased. Meanwhile, the expression of genes and proteins related to inflammation, such as E-sel, ICAM-1, VCAM-1, and MCP-1, decreased significantly, which inhibited the occurrence of inflammation (137). In ox-LDL-induced endothelial cells, kaempferol not only inhibited the PI3K/Akt/mTOR pathway, but also upregulated LC3-II/I and Beclin-1, which reduced endothelial cell apoptosis (138). Nevertheless, in a recent study, researchers applied kaempferol to atherosclerotic mice. It was a surprise to everyone that kaempferol inhibited inflammation and apoptosis by activating the membrane G-protein conjugated estrogen receptor (GPER), thereby activating the PI3K/AKT/Nrf2 signaling pathway (139). It’s revealed that when kaempferol acts on different models, its mechanism of action is different, but its preventive and therapeutic effects on atherosclerosis cannot be neglected. Besides, there are a variety of flavonols in fruit that have the same effect, as shown in Table 1.

**TABLE 1:** Flavonols derived from fruits are potential agents against Atherosclerosis.

Monomers	Source	Models	Mechanisms or effects	Chemical structure	References
Quercetin	Blueberry	Cholesterol induced Caco-2 cells and human embryonic kidney 293T cells Male Wistar rats fed with high cholesterol	↓ NPC1L1, total serum cholesterol		132
				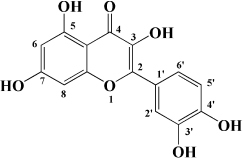	
		ApoE^–/–^ mice with high-fat diet	↑ IL-10, PPARγ, LXRα, ABCA1 ↓ TC, LDL-C, oxLDL, TNF-α, IL-6, PCSK9, CD36		133
		ox-LDL-Induced RAW264.7 Cells	↑ LC3-II/I, Beclin 1 ↓ MST1, Bcl-2, P21, P16		134
		High glucose induced human THP-1 monocytic cells	↑ Bcl-2 ↓ TNF-α, IL-1β, COX-2, CML, ROS, PKC, p47phox, p38, MAPK, PERK1/2, MAPK, NF-κB, RAGE		115
		ApoE^–/–^ mice fed with high-fat diet	↑ Sirt1 ↓ Slcam-1, IL-6, VCAM-1		135
Kaempferol	Filbert, grapes, strawberries, tomatoes, citrus fruits, apples, grapefruit	Copper-induced diluted plasma	↓ TBARS, MDA		136
				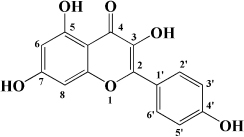	
		High-cholesterol-induced rabbits	↑ SOD ↓ TNF-α, IL-1β, MDA, E-sel, ICAM-1, VCAM-1, MCP-1,		137
		ox-LDL-induced HUVECs	↑ LC3-II/I, Beclin 1 ↓ p-Akt, p-mTOR		138
		HFD-OVX-induced APOE^–/–^ mice	↑ GPER, PI3K, Akt, Nrf2, SOD, GSH ↓ TCH-O, TG, LDL-C, HDL-C, MDA, TNF-α, IL-6, ICAM, VCAM		139
		Ox-LDL-induced HAECs	↑ GPER ↓ ROS		
Myricetin	Guava	ox-LDL-induced macrophages	↓ CD36-mediated ox-LDL uptake		140
				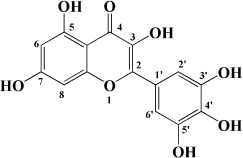	
		HASMCs and A7R5 cells	↓ CDK4, cyclin D3, MMP2, MMP9, TGFBR1, Smad2, Smad3		141
		ox-LDL-induced HUVECs	↑ miR-29a-3p ↓ GAS5, p-p65, p-IkBa, TLR4		142
Isorhamnetin	Sea buckthorn	Urotensin-II-induced primary VSMCs	↑ IL-10, MIF ↓ TNF-α, IL-1β, RhoA, ROCK II, ROCK I		143
				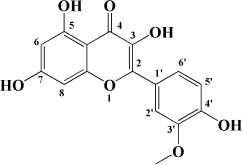	
		Ox-LDL-induced THP-1-derived macrophages	↑ MTP, AKT, HO-1 ↓ ROS, caspase 3, caspase 9, MPO, GSH-px, NOX		144
Galangin	Plantain	TNFα-induced HAECs	↓ E-selectin, intercellular adhesion molecule 1		145
				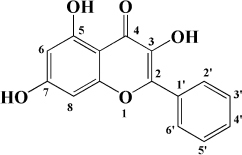	
Morin	Mulberry	ox-LDL-induced HUVECs	↑ p-AMPK ↓ ROS, MDA, SOD, IL-1β, IL-6, ICAM-1, VCAM-1, p-mTOR		146
				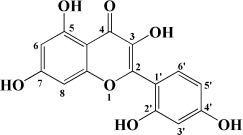	
		PDGF-induced VSMCs	↑ p27KIP1 ↓ CDK2, CDK4, cyclin D1, cyclin E, AKT, MMP, NF-κB, AP-1, Sp-1		147
Fisetin	Apple, persimmon, grape, strawberry	ApoE^–/–^ mice with high-fat diet	↑ SOD ↓ PCSK9, LOX-1, p53, p21, p16, ALT, AST, TC, LDL-C, VLDL-C, ox-LDL, MDA		148
				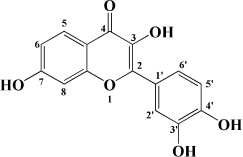	
		LPS-induced macrophages	↓ MCP-1, IL-1β, iNOS, NO, p-ERK, p-JNK, uPA, uPAR, MMP2, MMP9		149

### Flavone Glycosides

As an important component of flavonoids, the role of flavone glycoside in atherosclerosis has been gradually concerned. Rutin, a typical flavonoid glycoside found in apples, green tea and other sources, has antioxidant and anti-inflammatory activities and multiple therapeutic effects in atherosclerosis. In HUVEC cells induced by H_2_O_2_, rutin could enhance the expression and activity of eNOS by up-regulating the expression of basic fibroblast growth factor (bFGF), thereby increasing the production of NO and improving endothelial function (150). In high glucose induced VSMCs, rutin inhibited the migration and proliferation of VSMCs by inhibiting the MAPK (ERK1/2), BMK1, PI3K, and NF-κB signaling pathways (151). *In vivo*, when rutin was applied to streptozotocin (STZ)-induced ApoE^–/–^ mice, a significant reduction of atherosclerotic plaque in aorta was observed, accompanied by an increased proportion of VSMCs and enhanced plaque stability (152).

Naringin is the main compound of tomato, grapefruit, and related citrus. It is a flavanone glycoside with a disaccharide neohesperidose linked at C7 of the C6 (A ring)-C3 (C ring)-C6 (B ring) flavanone skeleton. It was first found in mice on a high-fat/high-cholesterol diet that treatment with naringin reduced plasma non-HDL cholesterol concentrations and ICAM-1, a biomarker of endothelial dysfunction. Transcriptome analysis of potential molecular targets suggested that the therapeutic effect of naringin may be related to its ability to reduce the adhesion of monocytes to endothelial cells and the proliferation of smooth muscle cells (153). In the following experiments, TNF-α-induced HUVECs were used as an *in vitro* model to further study the anti-atherosclerosis effect of naringin. The results showed that naringin inhibited the expression of adhesion molecules and chemokines, including VCAM-1, ICAM-1, and E-selectin, by inhibiting the activation of IKK/NF-κB signaling pathway (154). In addition, ox-LDL was used as a model drug to induce HUVECs. After naringin administration, VE-cadherin decomposition and F-actin remodeling were inhibited, and endothelial function was protected. At the same time, IL-1β, IL-6, IL-18, and other pro-inflammatory factors were decreased, and this protective effect was directly related to the YAP signaling pathway (155). In addition to these effects, naringin also has an ideal effect on regulating atherosclerosis through gut microbiota. Previous reports have found that naringin is highly hydrophilic and lacks the corresponding hydrolase in the body. This property protects naringin against digestion and absorption in the small intestine. Therefore, naringin can reach the colon and affect the composition of the gut microbiota after oral intake. The results showed that after naringin reached the colon, the relative abundance of g_*Bacteroides*, g_*Bifidobacterium*, and g_*Lactococcus* in the colon was decreased, and the content of bile salt hydrolyase was decreased. In contrast, the abundance of 7α-dehydroxylase producing bacteria-*Eubacterium*_fissicatena, *Eubacterium*_coprostanoligenes, and *Eubacterium*_brachy increased. The changes of the gut microbial community structure could directly promote the degradation of free bile acid, regulate the metabolism of cholesterol in the body, and increase the excretion of bile acid and neutral sterol by 1.6-fold and 4.3-fold, respectively. Cholesterol levels in serum and liver were also decreased to different degrees. These results suggested that naringin could alleviate atherosclerosis through the gut microbiome–liver–cholesterol axis (156). Other flavonoid glycosides are shown in Supplementary Table 2.

### Others

In addition to the above classification, there are many other classes of natural flavonoid compounds in fruits that can be used to treat atherosclerosis. For example, dihydromyricetin, derived from actinidia arguta, is structurally classified as a flavanones. In recent years, previous studies have found that dihydromyricetin can significantly improve hyperlipidemia in mice, reduce the levels of ox-LDL, IL-6, and TNF-α in serum, and restore inflammation to normal levels (173). Meanwhile, the protein expression of PPARα, LXRα, and ABCA1 was increased to promote lipid efflux and prevent lipid accumulation (173). In addition, *in vitro* cell models, dihydromyricetin could protect endothelial cell function, inhibit endothelial cell apoptosis, and prevent monocyte adhesion by activating Nrf2 or mir-21 signaling pathways (174, 175). Catechins derived from apples and peaches are flavanols that could reduce high glucose induced inflammation in human THP-1 cells through MAPK signaling pathway, reduce the expression of pro-inflammatory genes and proteins, including TNF-α, IL-1β, and COX-2, and reduce monocyte adhesion (115). Epicatechin, which also belongs to flavanols, is also distributed in apples, and has a good effect on severe atherosclerosis. Epicatechin attenuates the inflammatory process of atherosclerosis by inhibiting NF-κB signaling, reducing neutrophils and chemokines, and slowing stromal remodeling (176). In addition to these, anthocyanins such as pelargonidin, delphinidin, petunidin, xanthohumol, and phloretin in chalcone can also be used for the treatment of atherosclerosis, see Table 2 for details.

**TABLE 2:** Other flavonoids derived from fruits are potential agents against Atherosclerosis.

Classification	Monomers	Source	Models	Mechanisms or effects	Chemical structure	References
Flavanones	Dihydromyricetin	Actinidia arguta	HFD-induced atherosclerosis LDLr^–/–^ mice	↑ PPARα, LXRα, ABCA1 ↓ ox-LDL, IL-6, NOX2, TNF-α		173
					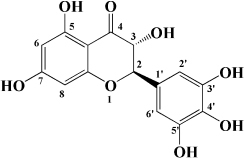	
			Palmitic acid-induced HUVECs	↑ Nrf2 ↓ LDH, IL-1β, caspase-1, ROS, mtROS		174
			Ox-LDL induced HUVECs and THP-1 cells	↑ NO, HDL, DDAH1-ADMA-eNOS ↓ VCAM-1, ICAM-1, E-Selectin, TG, LDL, TNF-α, IL-1β, IL-6, miR-21		175
Flavanol	Catechin	Peach, apple	High-glucose-induced human THP-1 cells	↑ Bcl-2 ↓ TNF-α, IL-1β, COX-2, CML, ROS, PKC, p47phox, p38, MAPK, p-ERK1/2, MAPK, NF-κB		115
					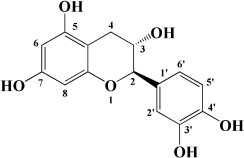	
	Epicatechin	Apple	Cholesterol-containing atherogenic diet fed ApoE*3-Leiden mice	↓ SAA, human-CRP, NF-κB		176
					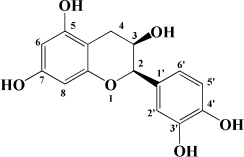	
Anthocyanidin	Pelargonidin	Acerola	PDGF-BB induced HASMCs	↑ F-actin ↓ FAK		177
					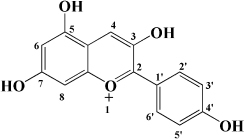	
	Delphinidin	Pitayas	OxLDL-induced HUVECs	↑ NO, Bcl-2 ↓ ROS, Bax		178
					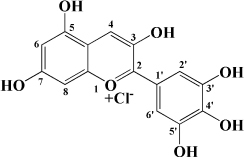	
			Serum and VEGF-induced BAECs	↑ ERK-1/-2, caveolin-1, p21^*WAF*1/*Cip*1^*CPSTABLEENTER*↓ RAS, cycoin D1		179
	Petunidin	Chokeberries	PDGF-BB-induced HASMC	↓ FAK, Akt, Src		180
					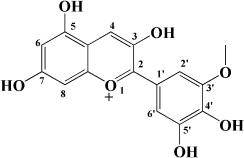	
Chalcone	Phloretin	Apple	High-glucose-induced HUVECs High-cholesterol diet and streptozotocin induced Apoe^–/–^ mice	↑ eNOS, KLF2 ↓ TG		181
					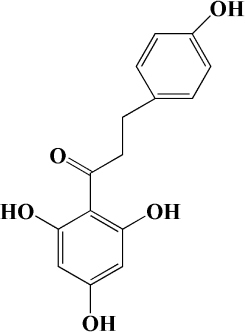	
			Thrombin-induced Human endothelial cells	↑ PAI-1 ↓ PAR-1, CD40, endothelial integrinβ3, P-selectin, CD40L, MCP-1, IL-6, IL-8, COX-2, PGE2		182
			PDGF-BB–induced RASMCs	↑ p27kip1 ↓ Akt, p38, CDK2, CDK4, p-Rb, VCAM-1, ICAM-1, MMP9, ROS		183
	Xanthohumol	Citrus	Western-type diet-fed ApoE^–/–^ mice	↑ AMP, CPT-1a, ABCG1 ↓ MCP-1, TC, FC, CE, SREBP-2		184
					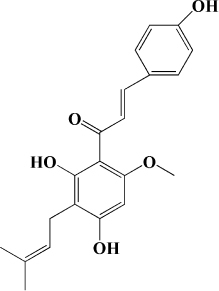	

## Conclusion and Perspectives

In recent years, atherosclerosis is increasingly threatening to human life, and the existing drugs or surgical treatments have certain limitations, so it is urgent to develop new drugs or treatment methods. Flavonoids are important bioactive components in fruits and are widely used in various nutritional products, cosmetics and medicines. At the same time, flavonoids from fruit have been shown to be effective in various stages of atherosclerosis development in recent studies. Based on our conclusion, current evidence suggests that fruit flavones have therapeutic effects on atherosclerosis by protecting endothelial cells, inhibiting foam cell formation, regulating lipid metabolism, and anti-inflammation, and the underlying molecular mechanisms are gradually being elucidated in more specific ways.

However, there are limitations and controversies that prevent the generalization of these results. It can be seen from Tables 1, 2 and Supplementary Tables 1, 2 that there are multiple *in vivo* and *in vitro* models to choose from in the study of flavonoids against atherosclerosis. When a compound acts on the same cell or animal stimulated by different modeling agents, its efficacy and mechanism of action will also be different, and an appropriate research model is an important prerequisite to ensure the accuracy of mechanism exploration and the reliability of results. At present, human or mouse cell lines are mostly used *in vitro* studies. For example, when studying the effect of flavonoids on inflammation in atherosclerosis, mouse leukemia cell lines RAW264.7 and J774 and human leukemia monocyte cell line THP-1 are mostly used, and THP-1 can differentiate into macrophages after the intervention of a variety of factors. However, in modern studies, it is generally believed that the atherosclerotic lesions in mice have been affected by microenvironmental factors, and the results of immortal cell lines do not reflect the *in vivo* process. Therefore, primary macrophages and peritoneal macrophages derived directly from animals have been applied in the study of flavonoid anti-atherosclerosis. *In vivo* models, researchers mostly use mouse and rabbit models as the main experimental platform. However, the major sites of atherosclerosis in humans are the coronary and carotid arteries, whereas in mice, the major sinus and innominate arteries are predominant. The *in vivo* model is also limited by the difference of lesion location.

All flavones from fruits have been studied in many aspects in clinical trials, but there is still a lack of clinical trials on atherosclerosis. At present, the research trend of flavonoids in fruits still remains to study their mechanism of action and molecular target, so as to explore their medicinal potential in atherosclerosis. However, whether a compound is suitable for development into a drug is also related to its bioavailability, metabolism, distribution, etc. As mentioned earlier, quercetin is an excellent potential drug with multiple therapeutic effects on atherosclerosis. When the solubility of quercetin was studied, it was found that the solubility of quercetin was 2.1 mg/L in water and 2 g/L in ethanol. This physical property directly limits the absorption of quercetin in the body (185). Pharmacokinetic results in human showed that the bioavailability of quercetin was very poor after a single oral administration. In addition, dietary quercetin is usually present in the form of glycosylation, which can be hydrolyzed by β-glucosidase in the digestive system and absorbed in the intestinal mucosa. Subsequently, quercetin can be transported to the liver *via* the portal vein and metabolized by glucuronidation, methylation, or sulfonylation (186). However, in recent studies, it was found that quercetin glucuronides, a major circulating metabolite, was rapidly eliminated in the human body, and the short elimination half-life was also an important reason for limiting the development of quercetin drugs (187). To solve the existing problems, the preparation of different delivery systems using nanotechnology has been widely accepted. For example, quercetin was encapsulated in nano-polymeric micelles, and then relevant pharmacokinetic experiments were performed in beagle dogs. The results showed that compared with free quercetin, the half-life of nano-quercetin was prolonged by 2.19 times after the application of nanotechnology, and its relative oral bioavailability was increased by 286%. Therefore, nanotechnology also has high potential in the treatment of atherosclerosis (188). In addition to polymer nanomaterials, inorganic nanomaterials, lipid-based nanomaterials, and biomimetic nanomaterials have been involved in the development of effective drugs for the treatment of atherosclerosis. Unfortunately, most drug development is still in the pre-clinical stage and has not been widely studied. In addition, the design of nanomaterials for dual therapy is also an important direction in future research.

Interest in the interaction of gut microbiota with flavonoids has increased in recent years. Under the action of intestinal flora, flavonoids can be hydrolyzed into aglycones in intestinal tract, and then reduced by hydrogenation of C ring. Finally, O-C2 bonds on C-ring are cleaved to form phenolic ketones and phenolic acids. In this process, the metabolic transformation of flavonoids enables them to be better absorbed by the small intestine and improve the bioavailability of flavonoids through systemic and local anti-atherosclerosis effects (189). At the same time, flavonoids in the intestinal tract can also affect the structure and function of gut microbiota, affecting the balance of gut microbiota (190). The interaction between flavonoids and gut microbiota provides a new perspective for understanding the effect mechanism of flavonoids on atherosclerosis. It is worth noting that up to now, the study on absorption, distribution and metabolism of flavonoids in the gut microbiota is still in its infancy. The therapeutic effect of flavonoid on atherosclerosis under the action of gut microbiota is not stable, and the underlying mechanism needs to be further explored.

Therefore, in future experiments, based on existing studies, we should increase the study of its pharmacokinetic, metabolic and pharmacodynamic characteristics *in vivo*, and find better flavonoid compounds and nanomaterials for the treatment of atherosclerosis, so as to find more reliable drugs for the treatment of disease.

## Author Contributions

R-LL and L-YW participated in the whole work. SL and H-XD participated in manuscript design, literature acquisition, and analysis. QZ and TZ participated in the drafting and revision of the manuscript. WP, YH, and CW gave final approval to the forthcoming edition, agreed on the journal to which the manuscript was submitted, and agreed to be responsible for all aspects of the work. All authors made a significant contribution to the work reported.

## Conflict of Interest

The authors declare that the research was conducted in the absence of any commercial or financial relationships that could be construed as a potential conflict of interest.

## Publisher’s Note

All claims expressed in this article are solely those of the authors and do not necessarily represent those of their affiliated organizations, or those of the publisher, the editors and the reviewers. Any product that may be evaluated in this article, or claim that may be made by its manufacturer, is not guaranteed or endorsed by the publisher.
