# Supplementary material for: Natural Flavonoids Derived From Fruits Are Potential Agents Against Atherosclerosis
Source: Front Nutr. 2022 Mar 24;9:862277. doi: 10.3389/fnut.2022.862277 (PMC8987282; doi:10.3389/fnut.2022.862277)
Supplement: Supplementary file 1 [file Table_1.doc]

**Table1. Flavones derived from fruits are potential agents against Atherosclerosis**

| **Monomers** | **Source** | **Models** | **Mechanisms or effects** | **Chemical structure** | **Refs.** |
| --- | --- | --- | --- | --- | --- |
| Apigenin | Orange, grapefruit and other fruits | SD rats with a high-fat diet | ↑ HDL-C, Bcl-2/Bax  ↓ TC, TG, LDC-c, LOX-1 |  | (100) |
|  |  | oxLDL-activated HUVECs | ↓ VCAM-1, E-selectin, LOX-1 |  | (101) |
|  |  | Glucose-induced HUVECs and HAECs | ↑ Bcl-2, AKT phosphorylation, eNOS, NO  ↓ PKCβⅡ, ROS, Bax, caspase-3, NF-кB |  | (102) |
|  |  | Trimethylamine-N-oxide-induced ISO-HAS human endothelial cells | ↓ LOX-1, NLRP3, TXNIP, MCP-1, VCAM-1, ICAM-1, CCL2, OLR1, SCARF1, CXCL16, PYCARD |  | (103) |
|  |  | H_2_O_2_-induced HUVECs | ↑ SOD, NO |  | (104) |
|  |  | RAW264.7 cells | ↑ ABCCA1  ↓ miR-33, TC, FC, CE |  | (105) |
|  |  | LPS-induced ApoE^-/-^ mice | ↑ ABCA1  ↓ miR-33, TLR-4, NF-кB p65 |  |  |
|  |  | LPS-induced RAW264.7 cells | ↓ TLR-4, MyD88, p-IKB-α, NF-кB p65 |  |  |
|  |  | Macrophages derived from C57BL/6 mice with western diet (containing 20% fat and 0.15% cholesterol) | ↑ Bax, cleaved caspase-3  ↓ AKT Ser473 phosphorylation, PAI-2, Mcl-1, Bcl-2 |  | (106) |
|  |  | oxLDL-induced macrophages | ↓ TNF-α, IL-1β, IL-6, Bcl-2  ↑ Atg/5Atg-7, Bax, Caspase-3 |  | (107). |
|  |  | LPS-induced macrophages and J774A.1 macrophages | ↓ IL-6, IL-1β, TNF-α, caspase-1, ERK1/2, NF-кB |  | (108) |
| Luteolin | Belimbi fruit and palm fruit | ApoE^-/-^ mice fed with high-fat diet | ↓ IL-6, TNF-α |  | (109) |
|  |  | oxLDL-induced primary mouse peritoneal macrophages | ↓ STAT3, IL-6, TNF-α, VCAM-1, ICAM-1 |  |  |
|  |  | oxLDL-induced RAW264.7 cells | ↑ Bax, Bak, cleaved caspase-9, cleaved caspase-3  ↓ Beclin-1, LC3-Ⅱ/LC3-Ⅰ |  | (110) |
|  |  | oxLDL-induced THP-1-derived macrophages | ↑ AMPK-SIRT1  ↓ TC, TG, LDC-c, IL-6, TNF-α, CD68, CCL2 |  | (111) |
|  |  | TNF-α-induced THP-1 and oxLDL-induced HUVECs | ↓ Integrin-β2, MMP-9, SR-A, SR-B1, PDGF-BB, occludin, PECAM-1 |  | (112) |
|  |  | H_2_O_2_-induced VSMCs | ↓ Scr, PDK1, Akt (308), Akt (473) |  | (113) |
|  |  | Angiotensin II- induced HUVECs | ↓ Scr, p-Akt (308), p-Akt (473) |  | (114) |
|  |  | LPS-induced RAW264.7 | ↑ IL-10, Arg-1  ↓ ROS, IL-1β, IL-18, iNOS, TNF-α, IL-6, MLRP3, ASC, caspase-1, Cox-2, X/XO, PGE2, HO• |  | (115,  116) |
|  |  | TNF-α-induced HUVECs | ↑ Bcl-2  ↓ ROS, Nox4, p22phox, ICAM-1, VCAM-1, caspase-3, caspase-9, ERK1/2 phosphorylation, NF-кB, p38 |  | (117) |
|  |  | oxLDL-activated HUVECs | ↓ VCAM-1, E-selectin, LOX-1 |  | (118) |
|  |  | Ad-TGFBR1 induced A7r5 and HASMC cell lines | ↓ TGFBR1/Smad, PCNA, Cyclin D1, MMP9, MMP2 |  | (119) |
|  |  | High glucose induced human THP-1 monocytic cells | ↑ Bcl-2  ↓ TNF-α, IL-1β, COX-2, CML, ROS, PKC, p47phox, pp38MAPK, PERK1/2 MAPPK, NF-кB, RAGE |  | (120) |
|  |  | Angiotensin II-induced macrophages of C57BL/6 mice | ↑ Bcl-2, caspase-3, Dectin-1, IL-10, Arg-1, CD206  ↓ Bax, cleaved caspase-3, IL-6, TNF-α, iNOS, CD16/32, PI3K/Akt |  | (121) |
|  |  | TNF-α-induced C57BL/6 mice | ↓ ICAM-1, VCAM-1, NF-кB, IKKβ, MCP/JE, CXCL1/KC ，IкBα |  | (122) |
| Tangeretin | tangerine | Hypercholesterolemia-induced rats | ↑ HDL  ↓ HMGCR, TC, LDL |  | (123) |
|  |  | (PDGF)-BB-induced rat aortic smooth muscle cells | ↑ p27^kip1^  ↓ PI3K/AKT, cyclin D1, cyclin E |  | (124) |
| Chrysoeriol | Euterpe oleracea Martius | PDGF-BB induced HASMCs | ↓ PDGF-Rβ, ERK1/2, p38, Akt |  | (125) |
| Nobiletin | Citrus Fruit | Lipoproteins-induced J774A.1 macrophages | ↓ acLDL, β-VLDL  ↑ SR-A |  | (126) |
|  |  | TPA-induced THP-1 human monocyte-like cells | ↓ ERK1/2, JNK1/2, Ser-63, AP-1, SR-A, SR-PSOX, CD36, CD68, Dil-acLDL |  | (127) |
|  |  | Ldlr^-/-^ mice fed with Western diet | ↑ LDLR, Pgc1a, Cpt1a  ↓ MTP, DGAT1/2, VLDL-TG |  | (128) |
|  |  | ox-LDL-induced-THP-1 | ↑ miR-590  ↓ IL-6, IL-1b, TNF-α, MCP-1 |  | (129) |
| Chrysin | Pyrus pashia | ox-LDL induced RAW264.7 macrophages | ↑PPARg, LXRa, ATP binding cassette, ABCG1  ↓SR-A1, SA-A2 |  | (130) |
|  |  | Human coronary artery endothelial cell | ↑KLF2  ↓miR-92a |  | (131) |
|  |  | Albino Wistar rats with an atherogenic diet | ↑ LPL, HMGCoA reductase, enzymatic, non-enzymatic antioxidants in hepatic  ↓ Lipid profile parameters, hepatic marker enzymes, MDA in serum |  | (132) |
|  |  | Metformin/acarbose-induced mice | ↓ TG, TNF-α, IL-1β |  | (133) |
| Acacetin | citrus fruits | High glucose- induced HUVECs | ↑ ATP, mitoBcl-2/mitoBax, NAD^+^, Sirt3, Sirt1, pAMPK, PGC-1α, SOD  ↓ ROS, MDA |  | (134) |
|  |  | STZ-induced ApoE^−/−^ mice | ↑ SOD, Bcl-2/Bax, PGC-1α, Sirt3, Sirt1, pAMPK |  |  |
|  |  | Ox-LDL induced EA.hy926 cells | ↑ HO-1, CAT, MsrA, Nrf2, Trx, SIRT1, SOD  ↓ ROS, MDA, keap |  | (135) |
|  |  | Western diet fed apoE^−/−^ mice | ↑ HDL-C, MsrA, Nrf2, PON1, ABCA1, SR-BI, ABCG1, CAT, IL-10  ↓ SAA, IL-6, TNF-α |  |  |
|  |  | Mouse macrophages J774.1 | ↑ ABCA1/G1, PPARγ mRNA, LXRα mRNA, CD36 mRNA, AMPK |  | (136) |

**Table2. Flavone glycosides derived from fruits are potential agents against Atherosclerosis**

| **Monomers** | **Source** | **Models** | **Mechanisms or effects** | **Chemical structure** | **Refs.** |
| --- | --- | --- | --- | --- | --- |
| Rutin | Grape | H_2_O_2_-induced HUVECs | ↑ NO, eNOS, bFGF |  | (155) |
|  |  | High-glucose-induced VSMCs | ↓ Phospho-p44/42 MAPK, phosphor-MEK1/2, phospho-PI3K, phospho-NF-κB, phospho-BMK1, ROS |  | (156) |
|  |  | Streptozotocin-induced ApoE^-/-^ mice | ↓ 3NT, 4HNE, p47phox, Nox4, MDA, ROS |  | (157) |
| Naringin | Tomatoes, grapefruits, and related citrus | wild-type mice fed a high-fat/high-cholesterol diet  apolipoprotein E-deficient mice fed a semisynthetic diet  TNF-α-stimulated HUVECs | ↓ VLDL, TC, sE-selectin, ICAM-1 |  | (158) |
|  |  | TNF-α-induced HUVECs | ↓ VCAM-1, ICAM-1, E-selectin, fractalkine/CX3CL1, MCP-1, RANTES, IKKα/β, IкB-α, NF-кB |  | (159) |
|  |  | ox-LDL-induced HUVECs | ↑ YAP  ↓ VE-cadherin disassembly, F-actin remodeling, IL-1β, IL-6, IL-18 |  | (160) |
|  |  | ApoE^−/−^ mice fed with a high-fat diet | ↑ 7α-dehydroxylase producing bacteria-*Eubacterium*_fissicatena, *Eubacterium*_coprostanoligenes and *Eubacterium*_brachy  ↓ Cholesterol in the serum and liver, g_*Bacteroides*, g_*Bifidobacterium* and g_*Lactococcus* |  | (161) |
| Mangiferin | Mango | LDLr^−/−^ mice | ↓ ROS, NADPH |  | (162) |
|  |  | Thioglycolate broth injected Wistar rats | ↑ TGF-β  ↓ 0_2_^-^, iNOS, TNF-α |  | (163) |
|  |  | Acetylated LDL-loaded RAW264.7 macrophages | ↑ ABCA1/G1, LXRα, PPARγ  ↓ LDL, TC, TG |  | (164) |
| Luteolin 7-glucoside | Fig | PDGF-BB-induced proliferation of VSMCs | ↓ ERK1/2, Akt, PLC-γ1 |  | (165) |
|  |  | TNF-α-induced HUVECs | ↓ ICAM-1, VCAM-1, NF-кB |  | (166) |
| Hesperidin | Citrus fruits | HFS-induced-LDLr^-/-^ mice | ↑ ABCG8, ABCA1, ABCG1  ↓ ACCα, FAS |  | (167) |
|  |  | Varenicline-induced-RAW 264.7  Varenicline-induced ApoE^-/-^ mice | ↑ ABCA1, ABCG1  ↓ CD36, LOX-1 |  | (168) |
| Cyanidin-3-O-glucoside | Strawberry, Blueberries | TNF-α-induced-HUVECs | ↑ SIRT1  ↓ miR-204-5p |  | (169) |
|  |  | AIN-93G diet fed ApoE-deficient mice | ↑ CYP7A1, LXRα |  | (170) |
|  |  | TNF-α-induced RASMCs | ↑ Caspase-3, caspase-9, Bax  ↓ Bcl-2, MMP-2, MMP-9, NF-кB, IкBα |  | (171) |
| Peonidin-3-O-β-glucoside | Grape | sCD40L-induced HUVECs | ↓ MMP-1, MMP-9, JNK, p38, VCAM-1, ICAM-1 |  | (172) |
| Delphinidin-3-Glucoside | Pitayas | OxLDL-induced HUVECs | ↑ NRFU, SIRT1, AMPK  ↓ ROS, O_2_^•-^, DYm |  | (173,174) |
| Orientin | Cucumber | High-glucose-induced HUVECs | ↓ ROS, CAM, MCP-1, IL-8, NF-кB |  | (175) |
|  |  | ox-LDLinduced RAW 264.7 | ↑ eNOS  ↓ IL-8, IL-6, CD36, ROS, angptl2, NF-кB, TNF-α |  | (176) |
| Vitexin | Passionflower | ApoE^-/-^ mice fed with Western diet | ↑ APEX1  ↓ SELE, CCL2, VCAM1, ROS, ICAM1, F4/80^+^ |  | (177) |
| Petunidin-3-glucoside | Chokeberries | TNF-α-induced HUVECs and THP-1 | ↓ VCAM-1, E-selectin, VEGF |  | (178) |
